# Homemade Food Allergen Extracts for Use in Skin Prick Tests in the Diagnosis of IgE-Mediated Food Allergy: A Good Alternative in the Absence of Commercially Available Extracts?

**DOI:** 10.3390/nu14030475

**Published:** 2022-01-21

**Authors:** Severina Terlouw, Frank E. van Boven, Monika Borsboom-van Zonneveld, Catharina de Graaf-in ‘t Veld, Marloes E. van Splunter, Paul L. A. van Daele, Maurits S. van Maaren, Marco W. J. Schreurs, Nicolette W. de Jong

**Affiliations:** 1 Department of Allergology, Albert Schweitzer Hospital, 3331 LZ Zwijndrecht, The Netherlands; m.vanzonneveld@asz.nl (M.B.-v.Z.); c.degraaf@asz.nl (C.d.G.-i.‘t.V.); 2 Internal Medicine, Allergology & Clinical Immunology, Erasmus MC, University Medical Centre, 3015 GD Rotterdam, The Netherlands; f.boven@erasmusmc.nl (F.E.v.B.); m.e.vansplunter@erasmusmc.nl (M.E.v.S.); p.l.a.vandaele@erasmusmc.nl (P.L.A.v.D.); m.vanmaaren@erasmusmc.nl (M.S.v.M.); n.w.dejong@erasmusmc.nl (N.W.d.J.); 3 Laboratory Medical Immunology, Department of Immunology, Erasmus Medical Centre, 3015 GD Rotterdam, The Netherlands; m.schreurs@erasmusmc.nl; 4 Department of Pediatric Allergology, Sophia Children Hospital, Erasmus Medical Centre, 3015 GD Rotterdam, The Netherlands

**Keywords:** diagnosis, extracts, food allergy, oral allergy syndrome, skin prick test, specific IgE

## Abstract

Introduction: The skin prick test (SPT) is the first step in the diagnosis of an immunoglobulin E (IgE)-mediated food allergy. The availability of commercial food allergen extracts is very limited, resulting in a need for alternative extraction methods of food allergens. The objective of this study was to compare the SPT results of homemade food allergen extracts with commercially available extracts. Methods: Adult patients with a suspected food allergy were included. Food allergen-specific symptoms were scored using a questionnaire. SPTs were performed with homemade and commercially available extracts (ALK-Abelló, Kopenhagen, Denmark) from almond, apple, hazelnut, peach, peanut, and walnut. Serum-specific IgE was measured with ISAC or ImmunoCAP™. Intra-class correlation coefficients (ICC) between the SPT results of both extract methods were calculated. The proportion of agreement with food allergen-specific symptoms was analyzed. Results: Fifty-four patients (mean age 36; range 19–69 years; female/male: 42/12) were included. The intra-class correlation coefficient (ICC) between the SPT results of both extract methods were strong for hazelnut 0.79 (*n* = 44) and walnut 0.78 (*n* = 31), moderate for apple 0.74 (*n* = 21) and peanut 0.66 (*n* = 28), and weak for almond 0.36 (*n* = 27) and peach 0.17 (*n* = 23). The proportion of agreement between SPT results and food allergen-specific symptoms was comparable for homemade and commercially available extracts, except for peach; 0.77 versus 0.36, respectively. Conclusion: In the diagnostic procedures to identify an IgE-mediated food allergy, homemade extracts from hazelnut and walnut appear to be a good alternative in the absence of commercially available food allergen extracts.

## 1. Introduction

The prevalence of food allergies in Europe is increasing rapidly. Currently, in adults, self-reported symptoms after ingesting different varieties of food are reported by 5.7–61.6% of people, and physician-diagnosed hypersensitivities are reported by 0.2–4.2% of people [1]. Diagnosing a suspected food allergy accurately is of great importance, both to prevent severe allergic reactions and to avoid unnecessary dietary restrictions caused by inaccurate diagnosis. The diagnosis of food allergy involves the use of skin prick tests (SPTs), allergen specific immunoglobulin E (sIgE), and oral food challenges (OFC) as a gold standard [2]. It is generally agreed that the core diagnostic step for type I mediated allergy, the SPT, should be further standardized, and further studies are necessary to define worldwide standards for allergen extracts [3]. In a recent EAACI position paper about in vivo diagnostic test allergens, the importance of reliable allergens was also stressed [4].

The evaluation of patients with a possible food allergy starts with an extensive food specific medical history and a physical examination. The focus should be on possible dietary triggers, the quantity and quality of the ingested food, possible facilitating co-factors around the time of the reaction (exercise, illness, use of medication), and the specific symptoms that led to the allergic reaction [5]. Knowledge of cross-reactivity within protein families would help to decide the ensuing pathway.

The next step in the diagnosis of a food allergy is measuring sensitization to the suspected food allergen by either performing an SPT with the suspected food allergen, and/or measuring serum sIgE. SPTs are a quick, reliable, and cheap method to measure sensitization. Although the negative predictive value (NPV) of SPTs often reaches 90% or more [5], false negative SPTs may occur if the used extracts are not standardized or have insufficient quantities of the allergen. In commercially available extracts of fruits and vegetables, the proteins might be destroyed during the manufacturing process, e.g., heating, giving less reliable results [6]. Generally, SPTs with food allergens have high sensitivity but low specificity, and must be interpreted with caution [6], and neither SPT nor sIgE are sufficient to diagnose food allergies on their own [7]. Soares-Weiser et al. (2014) also concluded that SPT and sIgE appear to be sensitive but not specific enough for diagnosing IgE-mediated food allergy, although this may differ between foods [8]. The availability of commercial food allergen extracts is limited, which leads to a need for alternative methods for the extraction of food allergens. One of the alternatives for commercial extracts might be to prepare homemade (HM) extracts through standardized protocols, but the quality of these extracts is unknown. Thus, the objective of this study was to compare SPT results of HM food allergen extracts and commercially available extracts.

## 2. Materials and Methods

### 2.1. Study Population

Adult patients with a suspected food allergy for at least one food allergen, visiting the outpatient clinic of the Allergology Department of the Albert Schweitzer hospital, were asked to participate in this study. The suspicion of food allergy was based on the patient’s clinical history and a physical examination by an allergist. All participants stopped their anti-histamines for at least 72 h before the SPT. Medical ethical approval was obtained for this study on 1 February 2018, trial number MEC-2017-486, NL61899.078.17. After patients signed an informed consent form, the inclusion took place from September 2018 until December 2020. A food -specific case history was conducted using an extensive food specific standardized questionnaire, which was filled out by the physician during the visit of the patient to the outpatient clinic. Symptoms were defined as the occurrence of oral itching, with or without angioedema of the lips and/or tongue (oral allergy syndrome [OAS]), respiratory symptoms, gastrointestinal symptoms (GI), and/or urticaria (skin symptoms). Inhalant allergies and concomitant medications used were reported. The PRACTALL score list was used to score symptoms and severity [2].

### 2.2. Skin Prick Tests

Based on the patient’s clinical history and the extensive food specific standardized questionnaire, the allergist chose a maximum of 4 food allergens, including the ones that were suspected as causing the food allergy. The SPTs with the chosen food allergens were performed with HM extracts as well as commercial extracts, both containing the same food allergen, in the same patient, at the same time. The food allergen extracts that were used in this study, available for both HM and commercial uses, were: almond, apple, hazelnut, peach, peanut and walnut. The SPT was conducted on the volar surface of the forearm by application of one drop of the allergenic extract to the skin. Subsequently, the dermis was punctured with a disposable standardized skin test needle (ALK-Abelló, Kopenhagen, Denmark), as recommended in the established EAACI guidelines [4]. Dilution buffer (ALK-Abelló, Kopenhagen, Denmark (nr. 002)) was used as a negative control. Mean values of two histamine dihydrochloride 10mg/mL (ALK-Abelló, Kopenhagen, Denmark (nr. 001))-induced wheal sizes were used as a positive control. To avoid puncture technical bias, the same nurse performed all SPTs. SPT results were obtained after 15 min; the contours of the allergen-induced wheal were encircled with a fine-tip pen and transferred to a record sheet by means of translucent tape (ALK-Abelló, Kopenhagen, Denmark). We compared the results of the Histamine Equivalent Prick result (HEP/PAAMOST) [9,10] of SPTs with HM extracts and ALK extracts. In addition to HEP measurement, the allergen-induced mean wheal diameter was measured to decide on positive and negative results (negative <3 mm Ø) according to the EAACI international guidelines [11].

### 2.3. HM Food Allergen Extracts

The raw material for each tested HM food allergen extract was carefully screened to select the material that best represented the allergen. Nuts and peanuts were fresh, not roasted and not salted, and were bought separately. The raw material was homogenized mechanically, ground with a mortar, and defatted with ether in a Soxhlet, air-dried, and stored at −20 °C until further use. Fruit and vegetables were bought fresh, and after being homogenized in a food processor, pulp was immediately stored in small portions for single use at −20 °C [10]. Pre-treated material of nuts and peanuts was defrosted and tested in a 5% or 10% extract in PBS (negative control; ALK-Abelló, Kopenhagen, Denmark (nr. 1036472)) as described by de Jong et al. [12]. In all cases, the allergens that were tested using HM extracts were compared with commercially available extracts from ALK-Abelló, Kopenhagen, Denmark; almond *Prunus dulcis* 1:20 m/V (nr. 764), apple *Pyrus Malus* spp. 1:20 W/V (nr. 658), hazelnut *Corylus avellana* 1:100 g/V (nr. 761), peach *Prunus persica* 1:20 G/V (nr. 613), peanut *Arachis hypogaea* 1:20 G/V (nr. 762), and walnut *Juglans regia* 1:20 W/V (nr. 766)).

### 2.4. Serum-Specific IgE

Serum-specific IgE levels were evaluated with the ImmunoCAP™ ISAC multiplex test, when available, or with the regular ImmunoCAP™ monoplex test, according to the manufacturer’s instructions (Thermo Fisher Scientific, Uppsala, Sweden). The result of ISAC multiplex sIgE was considered positive when ≥0.30 ISU. ImmunoCAP™ monoplex sIgE results were considered positive when ≥0.35 kU/L.

### 2.5. Statistical Analysis

Comparison of the SPT-HEP results of the HM and ALK extracts was performed by calculating the intra-class correlation coefficients (ICC) between the HEPs. These coefficients were considered very strong for ICC ≥ 0.9; strong for 0.75 ≤ ICC < 0.9; moderate for 0.5 ≤ ICC < 0.75; and weak for ICC < 0.5 [13]. SPT results were compared for almond, apple, hazelnut, peach, peanut, and walnut. We also compared the numbers of positive (≥3mm) SPT of HM and ALK extracts for the 6 food allergens. We compared the proportion of patients with a positive SPT of HM and of ALK using an exact binomial test. The agreement between qualitative SPT results (positive/negative) with symptoms per food allergen was assessed by calculating the proportion of patients with a positive SPT as well as any specific food allergen-related positive symptom, and a negative SPT with the absence of specific food allergen-related symptoms. Confidence intervals (CI) were calculated for these proportions, with a 0.05 level of significance. All calculations were performed by R (version 4.0.4 https://www.r-project.org, 16 December 2021).

Comparison of qualitative SPT results and sIgE results with food allergen-specific symptoms after consuming the specific food allergen was performed, and sensitivity, specificity, positive predictive value (PPV), negative predictive value (NPV), and the likelihood ratio were also reported.

## 3. Results

### 3.1. Study Population

Fifty-four adult patients (mean age 36; range 19-69 years) with a suspected food allergy were included. All participants reported one or more inhalant allergies: 40 (74%) to grass pollen, 51 (94%) to birch pollen, and 31 (57%) to house dust mites. Fifty participants (93%) reported OAS with or without GI symptoms, fourteen participants (26%) reported a skin reaction, and eighteen participants (33%) reported respiratory symptoms after ingestion of the suspected food allergen. Forty-six participants (85%) use any kind of anti-allergic medication. Of this group, forty-five (83%) use anti-histamines, seventeen (31%) use a nose spray, four (7%) use eye drops, thirteen (24%) use lung medication, and six (11%) of the participants need rescue medication (adrenalin). In Table 1, all patient characteristics are summarized.

The total numbers of patients that ever experienced symptoms after ingestion of the specific food allergen were: 16/27 for almond, 17/21 for apple, 36/44 for hazelnut, 16/23 for peach, 11/28 for peanut, and 22/31 for walnut. In total, forty SPTs (23%) were performed in patients who experienced no symptoms at all after consumption of the specific food allergen. Sixteen SPTs (9%) were performed in patients who could not answer the question as to whether they experienced symptoms after consumption of a specific food allergen, because they were on a strict diet free from the food for a long time, caused by, e.g., a positive sIgE in the past during routine testing. The results of any symptoms ever experienced after consumption of the specific food allergen gathered from the questionnaire are shown in Table 2.

### 3.2. Skin Prick Tests

One hundred and seventy-four SPTs were performed with the six included food allergens: 27 (16%) with almond, 21 (12%) with apple, 44 (25%) with hazelnut, 23 (13%) with peach, 28 (16%) with peanut, and 31 (18%) with walnut.

The mean HEP index with ALK food allergen extracts vs. HM extracts was; 0.96 vs. 0.51 for almond, 0.47 vs. 0.38 for apple, 1.40 vs. 1.61 for hazelnut, 0.11 vs. 0.83 for peach, 0.86 vs. 1.13 for peanut, and 0.42 vs. 0.39 for walnut, respectively. P-values for the comparison of the number of positive SPTs (≥ 3mm) were: 0.5 for almond, 0.5 for apple, 1.0 for hazelnut, <0.001 for peach, 0.63 for peanut, and 1.0 for walnut. The SPT-HEP results and ICC of the six food allergens are shown in Table 3.

**Figure 1:**
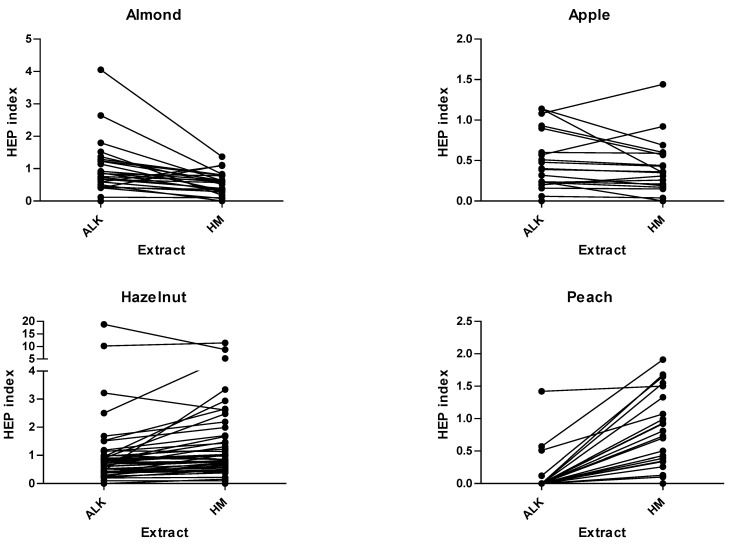

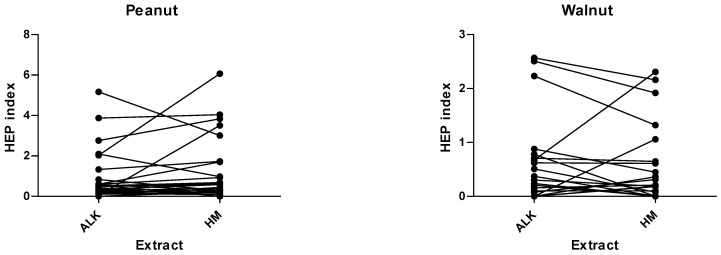
SPT-HEP results for ALK-HM regarding the 6 food allergens: almond, apple; hazelnut; figure peach; peanut; walnut.

The differences in the SPT-HEP results with both extracts are also depicted in Figure 1.

The ICCs between the SPT-HEP results for both extract methods, HM and commercial, were all significant; 0.36 (weak) for almond, 0.74 (moderate) for apple, 0.79 (strong) for hazelnut, 0.17 (weak) for peach, 0.66 (moderate) for peanut, and 0.78 (strong) for walnut.

### 3.3. Proportion of Agreement of SPT-HEP Results with Symptoms

**Figure 2:**
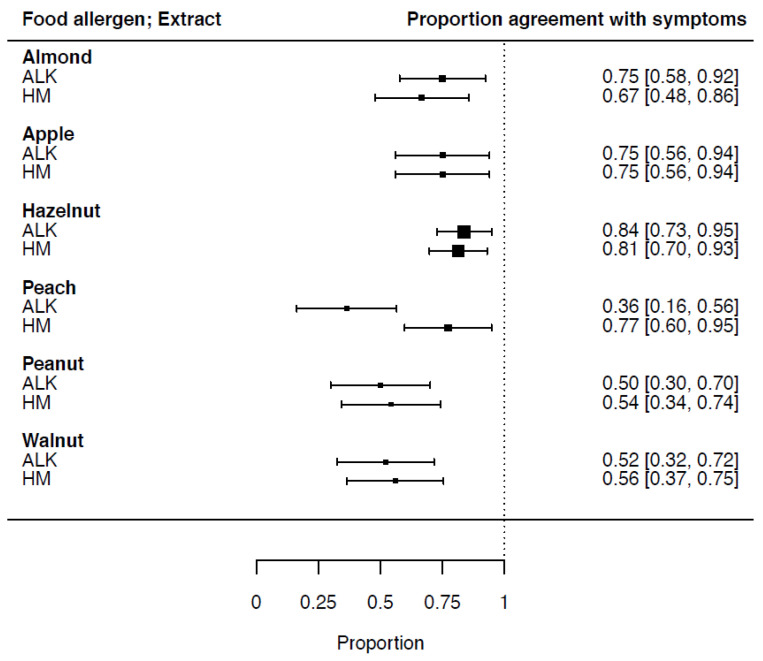
SPT-HEP results of ALK and HM extracts in relation to symptoms (proportion of agreement) and CI of 6 food allergens. ALK = Allergy Laboratories Kopenhagen, Denmark; HM = homemade.

Sensitization in relation to food-specific symptoms (proportion of agreement) and the confidence interval (CI) for ALK vs. HM extracts was calculated: for almond, 0.75 (CI 0.58–0.92) and 0.67 (CI 0.48–0.86), respectively; apple, 0.75 (CI 0.56–0.94) and 0.75 (CI 0.56–0.94), respectively; hazelnut, 0.84 (CI 0.73–0.95) and 0.81 (CI 0.70–0.93), respectively; peach, 0.36 (CI 0.16–0.56) and 0.77 (CI 0.60–0.95), respectively; peanut, 0.50 (CI 0.30–0.70) and 0.54 (CI 0.34–0.74), respectively; and walnut, 0.52 (CI 0.32–0.72) and 0.56 (CI 0.37–0.75), respectively. The sensitization in relation to the symptoms and CI of all six food allergen extracts is shown in Figure 2.

### 3.4. Serum-Specific IgE Measurements

Serum-specific IgE measured by ImmunoCAP™ (monoplex, Thermo Fisher Scientific, Uppsala, Sweden) for almond was positive in 10/27 cases. Specific IgE measured by ISAC (multiplex, Thermo Fisher Scientific, Uppsala, Sweden) was positive in all 42 sera. Specific IgE was positive in 18/20 for Mal d1 (apple), 40/42 for Cor a1 (hazelnut), 20/22 for Pru p1 (peach), 19/28 for Ara h8 (peanut), and 3/30 for Jug r 1 (walnut). The median and range of all sIgE measurements are shown in Table 4.

It appeared that other allergen components were only positive in a few cases; hazelnut Cor a8 and peach Pru p3 (both lipid transfer proteins [LTP]) were only positive in two and one patients, respectively, while major 2S albumins hazelnut Cor a 14 and peanut Ara h2 and Ara h6 were only positive in three, five and five cases, respectively. Proportion of agreement calculations were not feasible for these allergen components due to low power.

The proportion of agreement of specific IgE measurements in relation to symptoms and CI was calculated: for almond: 0.29 (CI 0.11–0.47); apple (Mal d1; PR10): 0.79 (CI 0.61–0.97); hazelnut (Cor a1; PR10): 0.80 (CI 0.68–0.93); peach (Pru p1; PR–10): 0.73 (CI 0.54–0.91); peanut (Ara h8; PR10): 0.71 (CI 0.53–0.89); and for walnut (Jug r1; 2S albumine): 0.21 (CI 0.05–0.37).

### 3.5. Accuracy of Sensitization Measurements in Relation to Reported Symptoms

Sensitivity and specificity measurements as well as the PPV, NPV and LR of SPT results in comparison to the reported symptoms were obtained (Table 5.) The mean sensitivity of SPT HM extracts and ALK extracts was 0.84 and 0.73, respectively. The mean specificity of SPT HM extracts and ALK extracts was 0.38 and 0.37, respectively.

## 4. Discussion

In this study, we compared SPT results for HM food allergen extracts with results for commercially available extracts, in patients with reported food-specific allergic symptoms, e.g., OAS, GI symptoms, skin symptoms, and/or respiratory symptoms, after ingestion of the suspected food. We performed SPTs in 54 patients, using both the HM extract and the commercially available extract of the same food allergen, within the same patient, at the same time. We found a strong correlation between both extract methods for hazelnut and walnut, moderate correlation for peanut and apple, and weak correlation for almond and peach. This indicates that these HM food allergen extracts are a good to moderate alternative in the absence of standardized commercially available extracts.

We found comparable sensitivity and specificity results for HM and ALK food allergen extracts. As expected, the mean sensitivity was high (0.84 and 0.73, respectively), but the specificity is considerably low for both extracts (0.38 and 0.37, respectively). Asero et al. described this low specificity earlier for food allergen extracts [14]. In particular, the hazelnut extract performed poorly in both extracts. One reason might be that we compared the results with doctor-diagnosed allergies without performing DBPCFCs. Another reason might be that most patients are sensitized to Cor a1, and consequently we lose these labile proteins during extraction. Interestingly, the sensitivity of the HM peach extract (0.94) performed very well, in contrast to the ALK extract, which has a sensitivity of 0.19. These results are in line with previous studies with fruit allergens, which point out that, in commercially available extracts of fruit and vegetables, the proteins may be destroyed during the manufacturing process [6]. A review by Foong and Santos in 2020 established higher sensitivity and specificity of SPTs with fresh fruit and vegetables, compared to commercial extracts, and acknowledged their importance in patients with pollen sensitization [15].

The considerable differences between SPT-HEP results of the HM and the commercially available extract of almond (mean HEP 0.51, range 0–1.37 vs. mean HEP 0.96, range 0–4.05, respectively) and the proportion of agreement for almond (0.67 vs. 0.75) must be seen in perspective. The sIgE measurements in relation to symptoms (proportion of agreement) and CI for almond (0.29 (CI 0.29–0.47)) were low. However, the perception of the patients with symptoms due to almond consumption can be argued; being sensitization to almond is often followed by a negative food challenge. In a cohort study by Arends et al., 189 almond challenges among a group of Dutch children were analyzed. A positive SPT with almond was found in 148 children (78%); 97/101 double blind placebo-controlled food challenges (DBPCFC) were negative [16].

In the 28 SPTs we performed with the peanut extract, we found 21 (75%) positive SPT(≥3mm) results with the commercially available extract vs. 23 (82%) with the HM extract (p-value 0.63). Thirteen patients (46%) could consume peanut without experiencing allergic symptoms. These outcomes were established in earlier studies; in 2005, Mortz et al. investigated the prevalence of peanut sensitization in an unselected population of adolescents and evaluated the clinical relevance of a positive sIgE or SPT to peanut, and the possible correlation between peanut and pollen sensitization. In a group of Danish adolescents, a peanut sensitization evaluated by ImmunoCAP™ and SPT of 5.8% and 3.4%, respectively, was found, while the point of prevalence of a peanut allergy, confirmed by oral challenge, was estimated to be 0.5%. Most peanut-sensitized adolescents had atopic diseases; intermittent allergic rhinitis was seen in 58–74%. The possibility of correlation between peanut and pollen (grass) sensitization was suggested [17]. Food challenge is still the gold standard for diagnosing food allergies, including suspected reaction to peanut [17,18,19,20].

HM food allergen extracts are prepared by standardized protocols. The HM allergen extracts of nuts and peanut are in all cases defatted during pre-processing. The removal of fat and oils, which are able to cause false positive type IV skin reactions, and other small particles, e.g., minerals, improves the exposure of allergenic proteins and extraction efficiency, and removes components that are insoluble in water [12,21].

Defatted and dried HM allergen material (dry powder) can be stored at −20 °C, which improves the long-term stability. De Jong et al. showed good stability results with the same method (HM), comparing fresh, 3-month-old, and 6-month-old extracts. In this earlier study, batch-to-batch comparisons with coriander, hazelnut, peach, and sesame seed gave coefficients of variation of 39%, 33%, 37%, and 26%, respectively. Overall, pair wise comparison of dose response SPT results with the four different HM extracts using 5%, 10%, and 20% were significant in all cases [12]. Secondly, the HM extracts appeared to be safe, as no adverse events occurred in the 2004 study, as well as in the current study. Finally, the method of preparing HM extracts is clearly extremely cost-effective. An analyst can prepare the material in the hospital laboratory, using food from the local grocery, and even more rare sources (e.g., new food sources such as seaweed, tropical fruits such as papaya, and new legumes such as lentils) can be extracted easily at a low cost.

There are some limitations to this study: first, most patients included in this study suffer from an inhalant allergy (sensitization birch pollen: 94%, grass pollen: 74%). We did not specifically select these patients, but as we performed the study in a peripheral hospital (second line), the population differs from, e.g., an Academic Center. Consequently, the suspected food allergy in these patients was most likely caused by cross reactivity, which could be confirmed by a high percentage of sensitization to several PR-10-specific allergens (Cor a 1, Ara h 8, Pru p 1, Mal d 1). This might cause some bias, as we therefore did not test the allergen extracts in patients with a primary food allergy. The low sIgE found for LTP proteins (Cor a8, Ara h9, Jug r3, and Pru p3) confirms the population of the included patients. Consequently, the proportion of agreement for walnut Jug r1 is low (0.24). Finally, in a peripheral hospital, we did not perform the gold standard for the diagnosis of food allergy; the DBPCFC. Comparing SPT results with suspected food allergy is not in accordance to the guidelines, but must be seen as a first step in the diagnosis of a food allergy [2].

## 5. Conclusions

In this study, we found that the SPT-HEP results of the HM extracts are comparable with the SPT-HEP results of commercially available extracts for hazelnut and walnut, and moderately comparable for apple and peanut. We recommend further studies with HM extracts of food allergens in another population, e.g., children and patients with a primary food allergy. Furthermore, we also recommend the characterization and identification of allergenic proteins in HM food allergen extracts. Commercial food allergen extracts will be less available in the near future, caused by new European government regulations. Developing and validating educational tools on how to produce suitable and reproducible HM food allergen extracts will increase the establishment of vertical and horizontal networks between Academic Centers of excellence, allergy specialists, and primary health care practitioners [22]. These developments will increase the knowledge, quality, and use of HM food allergens extracts, and might be one step forward in the complex diagnosis of food allergies.

## Figures and Tables

**Table 1:** Patient characteristics.

Patient Characteristics		
Numbers included	54	
Female/male	42	12
Mean age/range	36	19–69
	*n*	%
Inhalant allergy	54	100
Grass pollen	40	74
Birch pollen	51	94
House dust mite	31	57
Pets	33	61
Medication used	46	85
Anti-histamines	45	83
Corticosteroid nose spray	17	31
Eye drops	4	7
Lung medication	13	24
Adrenaline	6	11
Food allergy symptoms		
GI + OAS	50	93
Skin	14	26
Lung	18	33

*n* = number; GI = gastro-intestinal symptoms; OAS = oral allergy symptoms; Lung = respiratory symptoms; Skin = skin symptoms.

**Table 2:** Symptoms per food allergen.

Symptoms Per Food Allergen							
	Almond	Apple	Hazelnut	Peach	Peanut	Walnut	Total
*n* (%)	27 (16)	21 (12)	44 (25)	23 (13)	28 (16)	31 (18)	174 (100)
	*n* (%)	*n* (%)	*n* (%)	*n* (%)	*n* (%)	*n* (%)	*n* (%)
Consuming/	8 (30)	3 (14)	7 (16)	6 (26)	13 (46)	3 (10)	40 (23)
no symptoms							
NA (strict diet)	3 (11)	1 (5)	1 (2)	1 (4)	4 (14)	6 (19)	16 (9)
Symptoms:							
GI/OAS	12 (44)	12 (57)	23 (52)	12 (52)	5 (18)	14 (45)	78 (45)
Skin	0	0	1 (2)	0	1 (4)	0	2 (1)
Lung	1 (4)	0	0	0	0	0	1 (1)
GI/OAS + Skin	1 (4)	1 (5)	2 (5)	1 (4)	0	1 (3)	6 (3)
GI/OAS + Lung	2 (7)	3 (14)	8 (18)	3 (13)	2 (7)	5 (16)	23 (13)
GI/OAS + Skin + Lung	0	1 (5)	2 (5)	0	2 (7)	2 (6)	7 (4)
Skin + Lung	0	0	0	0	1 (4)	0	1 (1)

SPT = skin prick test; *n* = number; NA = not applicable because of patient on a strict diet free from the food allergen; GI = gastro-intestinal symptoms; OAS = oral allergy syndrome; skin = skin symptoms; lung = respiratory symptoms.

**Table 3:** Skin prick test results per food allergen.

SPT Results Per Food Allergen								
		Almond	Apple	Hazelnut	Peach	Peanut	Walnut	Total
ALK	Positive ≥3 mm	24	19	41	4	21	14	123
	%	89	90	93	17	75	45	71
	Mean HEP index	0.96	0.47	1.40	0.11	0.86	0.42	
	Range HEP index	0–4.05	0–1.14	0–18.85	0–1.42	0–5.17	0–2.57	
HM	Positive ≥3 mm	22	17	42	20	23	14	138
	%	81	81	95	87	82	45	79
	Mean HEP index	0.51	0.38	1.61	0.83	1.13	0.39	
	Range HEP index	0–1.37	0–1.44	0–11.44	0–1.91	0–6.07	0–2.31	
ICC		0.36	0.74	0.79	0.17	0.66	0.78	
95% CI for ICC		0 to 0.65	0.47 to 0.89	0.65 to 0.88	0 to 0.49	0.39 to 0.82	0.59 to 0.89	
*p*-value HEP		0.03	˂0.0001	˂0.0001	0.015	˂0.0001	˂0.0001	
Strength of ICC		weak	moderate	strong	weak	moderate	strong	

SPT = Skin Prick Test; *n* = number; HEP = Histamine Equivalent Prick test; ALK = Allergy Laboratories Kopenhagen, Denmark; HM = homemade; ICC = intra-class correlation coefficient.

**Table 4:** Serum specific IgE measurements.

Serum Specific IgE Measurements					
		*n* =	Positive *n* =	Mean ISU	Range ISU
Almond *		27	10	0.60 *	0.0–2.98 *
Apple	Mal d1	20	18	15.38	0–64.1
Hazelnut	Cor a1	42	40	7.42	0–31.2
	Cor a8	42	2	1.34	0–54.4
	Cor a9	42	2	0.30	0–9.83
	Cor a14	42	3	3.03	0–105.6
Peach	Pru p1	22	20	7.50	0–60.9
	Pru p3	22	1	0.23	0–4.67
Peanut	Ara h2	28	5	1.56	0–20.3
	Ara h6	28	5	1.15	0–14.4
	Ara h8	28	19	2.88	0–14.0
	Ara h9	28	3	3.39	0–8.29
Walnut	Jug r1	30	3	2.41	0–65
	Jug r3	30	0	0	0

*n* = number; * = measured by ImmunoCAP™ Thermo Fisher Scientific, Uppsala, Sweden

**Table 5:** Accuracy of sensitization measurements in relation to reported symptoms.

Accuracy of Sensitization Measurements in Relation to Reported Symptoms							
Extract		Sensitivity	Specificity	PPV	NPV	LR+	LR−
Almond	ALK	1.00	0.25	0.73	1.00	1.33	0.00
	HM	0.88	0.25	0.70	0.50	1.17	0.50
	sIgE Almond	0.44	0.20	0.25	0.38	0.56	2.78
Apple	ALK	0.88	0.00	0.83	0.00	0.88	NA
	HM	0.82	0.33	0.88	0.25	1.24	0.53
	Mal d1	0.88	0.00	0.88	0.00	0.88	NA
Hazelnut	ALK	0.97	0.25	0.85	0.67	1.30	0.11
	HM	0.97	0.13	0.83	0.50	1.11	0.23
	Cor a1	0.82	0.50	0.97	0.13	1.64	0.36
Peach	ALK	0.19	0.83	0.75	0.28	1.13	0.98
	HM	0.94	0.33	0.79	0.67	1.41	0.19
	Pru p1	0.75	0.50	0.94	0.17	1.50	0.50
Peanut	ALK	0.82	0.23	0.47	0.60	1.06	0.79
	HM	0.91	0.23	0.50	0.75	1.18	0.39
	Ara h2	1.00	0.65	0.36	1.00	2.86	0.00
	Ara h8	0.53	0.71	0.82	0.38	1.85	0.66
Walnut	ALK	0.50	0.67	0.92	0.15	1.50	0.75
	HM	0.50	1.00	1.00	0.21	NA	0.50
	Jug r1	1.00	0.14	0.10	1.00	1.16	0.00

ALK = Allergy Laboratories Kopenhagen, Denmark; HM = homemade; PPV = positive predictive value; NPV = negative predictive value; LR+ = positive likelihood ratio; LR− = negative likelihood ratio; NA = not applicable.

## Data Availability

The original database is not available online.

## Abbreviations

ACE	Academic Center of Excellence
ALK	Allergy Laboratories Kopenhagen
CI	confidence interval
DBPCFC	double blind placebo controlled food challenge
EAACI	European Academy of Allergy and Clinical Immunology
e.g.,	exempli gratia
F	female
FN	false negative
FP	false positive
HEP	Histamine Equivalent Prick test
HM	homemade
ICC	intra-class correlation coefficient
IgE	immunoglobulin E
ISAC	immuno-solid phase allergen chip
LR	likelihood ratio
LTP	lipid transfer proteins
M	male
Mm	millimetre
n	number
NA	not applicable
NPV	negative predictive value
Nr	number
OAS	oral allergy syndrome
OFC	oral food challenge
PPV	positive predictive value
Resp	respectively
Sens	sensitivity
sIgE	specific immunoglobulin E
Spec	specificity
SPT	skin prick test
TN	true negative
TP	true positive
vs.	versus
